# Mapping the ethical landscape of digital biomarkers: A scoping review

**DOI:** 10.1371/journal.pdig.0000519

**Published:** 2024-05-16

**Authors:** Mattia Andreoletti, Luana Haller, Effy Vayena, Alessandro Blasimme

**Affiliations:** Department of Health Sciences and Technology, ETH Zurich, Zurich, Switzerland; McGill University, CANADA

## Abstract

In the evolving landscape of digital medicine, digital biomarkers have emerged as a transformative source of health data, positioning them as an indispensable element for the future of the discipline. This necessitates a comprehensive exploration of the ethical complexities and challenges intrinsic to this cutting-edge technology. To address this imperative, we conducted a scoping review, seeking to distill the scientific literature exploring the ethical dimensions of the use of digital biomarkers. By closely scrutinizing the literature, this review aims to bring to light the underlying ethical issues associated with the development and integration of digital biomarkers into medical practice.

## Author summary

This scoping review focuses on the ethical complexities inherent in the use of digital biomarkers, recognizing the necessity of understanding and addressing these challenges. The review examines literature across various fields to illuminate the ethical issues surrounding the development, validation, and implementation of digital biomarkers. The following ethical concerns are highlighted: privacy and data security, informed consent, validation, equity and sustainability, transparency, impact of stigmatization, regulatory issues, accountability, and data ownership.

## 1. Introduction

Roughly speaking, digital biomarkers are biological traits that can be measured using digital devices (see below). In recent years, the use of novel digital biomarkers in both research and clinical settings has sparked significant scientific interest, due to their potential to revolutionize health monitoring, diagnosis, biomedical discovery, and drug development. Considerable effort is underway to develop and validate digital biomarkers in diverse domains such as neurology, mental health, cardiology, and healthy aging. Alongside the development of such digital tools, there is a growing focus on the ethical considerations associated with these advancements.

The rise of digital biomarkers has been facilitated by the widespread adoption of smartphones, wearable devices, and Internet of Things (IoT) technologies, which enable extensive data collection and real-time analysis. These digital tools have empowered individuals to actively participate in their own healthcare management by providing access to personalized health information and facilitating early detection and prevention of disease. Digital biomarkers extend beyond the direct measurement of pathophysiological variables, to encompass an array of digital signals related to medical conditions or health trajectory. For instance, digital biomarkers such as key-stroke dynamics can correlate to fluctuations in individual cognitive capacity, enabling real-time detection of functional impairment caused by stress, anxiety, fatigue, or an underlying neurological condition [1].

Unlike molecular biomarkers, digital biomarkers are not primarily intended as tools for patient stratification. Digital biomarkers have capacity to measure proxies of both clinical and pre-clinical conditions, and detect pre-clinical signs of functional deterioration. Thus, they hold promise to greatly expand our ability to monitor individual health across the life course, and are pivotal to enabling preventive, health promotion and public health interventions, including those outside of conventional clinical settings; for instance, in the field of healthy aging. In this respect, digital biomarkers represent a major component to the success of digital health.

Given the disruptive potential of digital biomarkers, it is crucial to undertake a comprehensive exploration of the ethical considerations and challenges associated with their implementation.

An example of a digital biomarker is variability in gait speed, measured by wearables, for early detection of Alzheimer’s Disease (AD). Alzheimer’s disease patients commonly display motor difficulties in the initial phases of the condition, occurring a minimum of ten years before cognitive impairment symptoms become evident [2]. Motor difficulties can be actively assessed in a gait laboratory using tests such as walking for a predetermined distance or duration, or continuously distant monitored with digital devices. However, the collection of such data raises immediate ethical concerns, including ensuring proper informed consent, determining data access rights, outlining usage protocols, implementing robust security measures, and delineating constraints on data accessibility–just to name a few.

The use of a scoping review methodology in this study enables a thorough examination of the ethical landscape pertaining to digital biomarkers. This approach facilitates a comprehensive exploration of ethical considerations and challenges that arise during their implementation, providing a general understanding of ethical challenges in this space. Overall, our research endeavors to answer the following question: What ethical issues are associated with the development, validation, and use of digital biomarkers? By employing a scoping review methodology, we can map the breadth of available evidence, encompassing studies in different fields. Through this approach, we aim to identify key ethical issues, address gaps in the existing literature, and shed light on potential areas of concern, ultimately contributing to the development of robust ethical frameworks and policies guiding the responsible development and use of digital biomarkers.

## 2. Background

### 2.1 Definition

Biomarkers are indicators of biologic processes, pathologic processes, or biological reactions to a therapeutic intervention [3]. Initially, biomarkers were primarily used for patient stratification in the context of clinical research, as they enable prognosis, and can predict which patients are more likely to respond well to a drug and experience fewer side effects. Biomarkers have been widely adopted in drug development to measure drug response in terms of pharmacodynamics, target engagement, safety, and toxicity. More recently, biomarkers have found application in the clinical context, most notably in oncology, where they have enabled considerable progress in targeted oncological therapy [4] through the development of companion diagnostics assays [5,6].

The first occurrences of the term *digital* biomarker in the literature can be found in two studies from the mid 2010s, one describing a patch that continuously monitors oxygen saturation during sleep, to detect sleep respiratory problems [7]; the other, illustrating the use of thoracic impedance measurements to identify high-risk patients in the setting of developing drugs for cardiovascular conditions [8]. It is worth noting that the concept of digital biomarkers has since evolved significantly.

The term "digital" refers to the use of numerous types of hardware, sensors, and software to collect, analyze and present data. Measurements frequently take place in home environments and can involve both commercial products such as smartphone and fitness bands, and medical devices such as wearables, and implantable or ingestible sensors [9]. The prospects of using large arrays of digitally collected data for health-related predictions and to inform clinical decision making, are also linked to the use of advanced data analytics techniques such as machine learning, deep neural networks, natural language processing and predictive analytics [10].

The notion of digital biomarker can also be used to refer more generally to the collection of any health-relevant data enabled by digital devices (see e.g., [11]). Here we distinguish digital biomarkers from digital phenotyping, defined as the collection and analysis of contextual digital data through personal devices and digital platforms (smartphone usage patterns, social media activity, GPS location) to infer an individual’s behavioral or psychological characteristics, either in isolation [12] or in conjunction with clinical measurements, such as health records and molecular or imaging data [13]. Digital phenotyping tends to emphasize behavioral and lifestyle aspects, but can also inform diagnosis of specific disorders [14].

On the other hand, digital biomarkers most often refer to objective, quantifiable measures obtained through digital devices or sensors beyond those that are commercially available, providing information about an individual’s pathological state. Digital biomarkers tend to focus on physiological signals and pathophysiological processes, enabling more direct inference of health outcomes. There is considerable semantic overlap between the two approaches, with both offering great promise for early detection, monitoring, and personalized interventions in healthcare.

With discussion of definitions ongoing, some authors have advocated for a more stringent and consistent usage of the term digital biomarker (see e.g., [15]), in contrast to its current varied application. One recent attempt at clarification was made by the FDA, defining a digital biomarker as “a characteristic or set of characteristics, collected from digital health technologies, that is measured as an indicator of normal biological processes, pathogenic processes, or responses to an exposure or intervention, including therapeutic interventions” [16]. This definition has the merit of clearly establishing the scope of digital biomarkers and highlighting their potential application in diagnostics, pharmacodynamics/response, and monitoring. It can serve as a crucial entry point for discussion of this topic, including ethical considerations.

## 3. Methodology

### 3.1 Information sources and eligibility criteria

The methodology for this scoping review study was developed based on the Arksey and O’Malley framework [17]. To ensure comprehensive coverage of the literature, we conducted our search across three major electronic databases: PubMed, Scopus, and Web of Science. This multi-platform approach aimed to minimize the risk of missing relevant papers. Throughout the screening process, we independently reviewed the papers to ensure an unbiased approach, applying eligibility and exclusion criteria, and discussing each point of disagreement.

**Fig 1:**
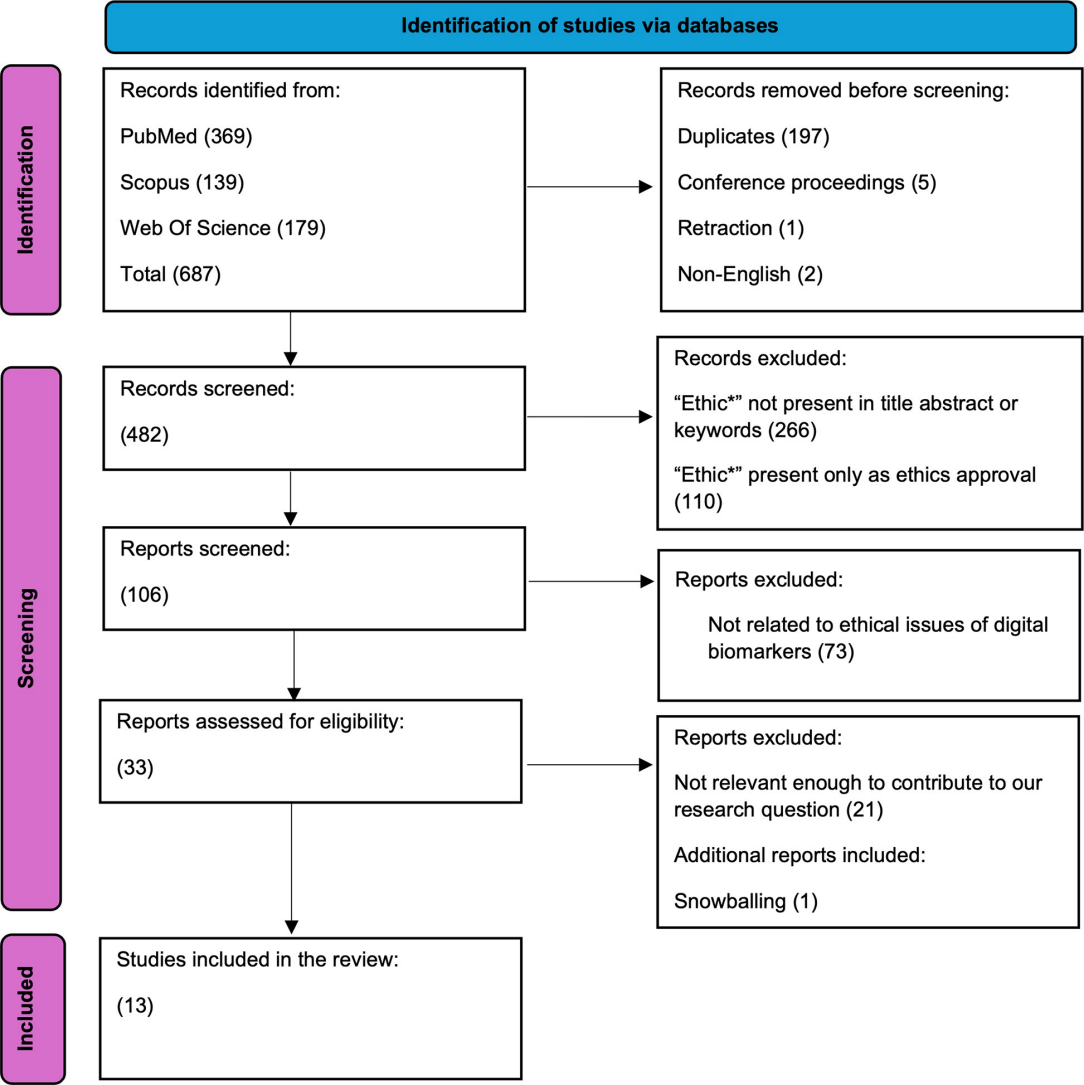
Scoping Literature Review flow chart (PRISMA).

Considering the relatively recent emergence of the field, we set the timeframe for the literature search from 2007 (the year of the first iPhone release) to 2022. This timeframe was chosen to ensure replicability and establish a library that can be easily updated in the future. The final search string used was: (digital OR sensor* OR mobile OR smartphone OR biometr* OR wearable) AND (biomarker*) AND ethic* in title, abstract, and keywords. We used the most common synonyms and related keywords for digital, in order to capture a larger sample of the literature. We considered papers published in peer-reviewed journals and book chapters. We also considered review articles, given the limited availability of original research. Articles had to be written in English for inclusion in our sample (Fig 1).

Our literature search was conducted between March and April 2023. Search results from all databases yielded a total count of 687 articles, which were then exported into EndNote software for sorting and removal of duplicates. After eliminating duplicates (197), retractions (1), conference proceedings (5), and non-English literature (2), our library consisted of 482 records.

These records were initially screened for the presence of the keyword "ethic(s)" in the title, abstract, or keywords. Abstracts mentioning ethics solely in the context of ethics dissemination or approval by an Ethics Committee were excluded. We revisited 106 papers and removed those that were not relevant to ethical issues of digital biomarkers, based on a more thoughtful analysis of the abstracts. Any inconsistencies were resolved through joint examination of the full text. This further screening process resulted in 33 records eligible for inclusion. Additionally, snowballing research led to the identification of one more relevant publication. After this selection process, a total of 13 papers were included in this review (Table 1).

**Table 1:** Details of the articles included in the review.

Authors	Title	Publication Venue	Area	Type
Bourla et al. 2018	Psychiatrists’ Attitudes Toward Disruptive New Technologies: Mixed-Methods Study	JMIR Mental Health	Mental Health/Psychiatry	Original research article
Chen et al. 2022	Development of Digital Biomarkers of Mental Illness via Mobile Apps for Personalized Treatment and Diagnosis	Journal of Personalized Medicine	Personalized and Digital Medicine	Review
Coravos et al.2019	Digital Medicine: A Primer on Measurement	Digital Biomarkers	Personalized and Digital Medicine	Review
Dagum & Montag 2022	Ethical Considerations of Digital Phenotyping from the Perspective of a Healthcare Practitioner	Digital Phenotyping and Mobile Sensing: New Developments in Psychoinformatics (edited by H. Baumeister and C. Montag)	Mental Health/Psychiatry	Book chapter
Fröhlich et al. 2022	Leveraging the Potential of Digital Technology for Better Individualized Treatment of Parkinson’s Disease	Frontiers in Neurology	Neurology	Review
Gold et al. 2018	Digital technologies as biomarkers, clinical outcomes assessment, and recruitment tools in Alzheimer’s disease clinical trials	Alzheimer’s & Dementia: Translational Research & Clinical Interventions	Mental Health/Psychiatry	Perspective
Josephy-Hernandez et al. 2021	Survey on Acceptance of Passive Technology Monitoring for Early Detection of Cognitive Impairment	Digital Biomarkers	Personalized and Digital Medicine	Original research article
Low 2020	Harnessing consumer smartphone and wearable sensors for clinical cancer research	NPJ Digital Medicine	Personalized and Digital Medicine	Review
Parziale & Mascalzoni 2022	Digital Biomarkers in Psychiatric Research: Data Protection Qualifications in a Complex Ecosystem	Frontiers in Psychiatry	Mental Health/Psychiatry	Review
Sim 2019	Mobile devices and health	New England Journal of Medicine	Medicine (general)	Review
Slavich et al. 2019	Stress measurement using speech: Recent advancements, validation issues, and ethical and privacy considerations	Stress	Mental Health/Psychiatry	Commentary
Smith et al. 2021	Affective Computing for Late-Life Mood and Cognitive Disorders	Frontiers in Psychiatry	Mental Health/Psychiatry	Review
Whelan et al. 2022	Developments in scalable strategies for detecting early markers of cognitive decline	Translational Psychiatry	Mental Health/Psychiatry	Review

The full text of each selected paper was then thoroughly analyzed to identify all ethical issues related to digital biomarkers. Articles that did not explicitly discuss ethical issues associated with digital biomarkers, even in a limited manner, were excluded. To enhance comprehension of the wide-ranging areas explored in the chosen papers, we systematically categorized the papers based on their respective topics. This approach enabled us to construct a comprehensive overview of the diverse domains and subtopics addressed in the literature. Significant findings from each paper were then succinctly summarized in an overview table, streamlining the subsequent analysis of the results.

## 4. Results

**Fig 2:**
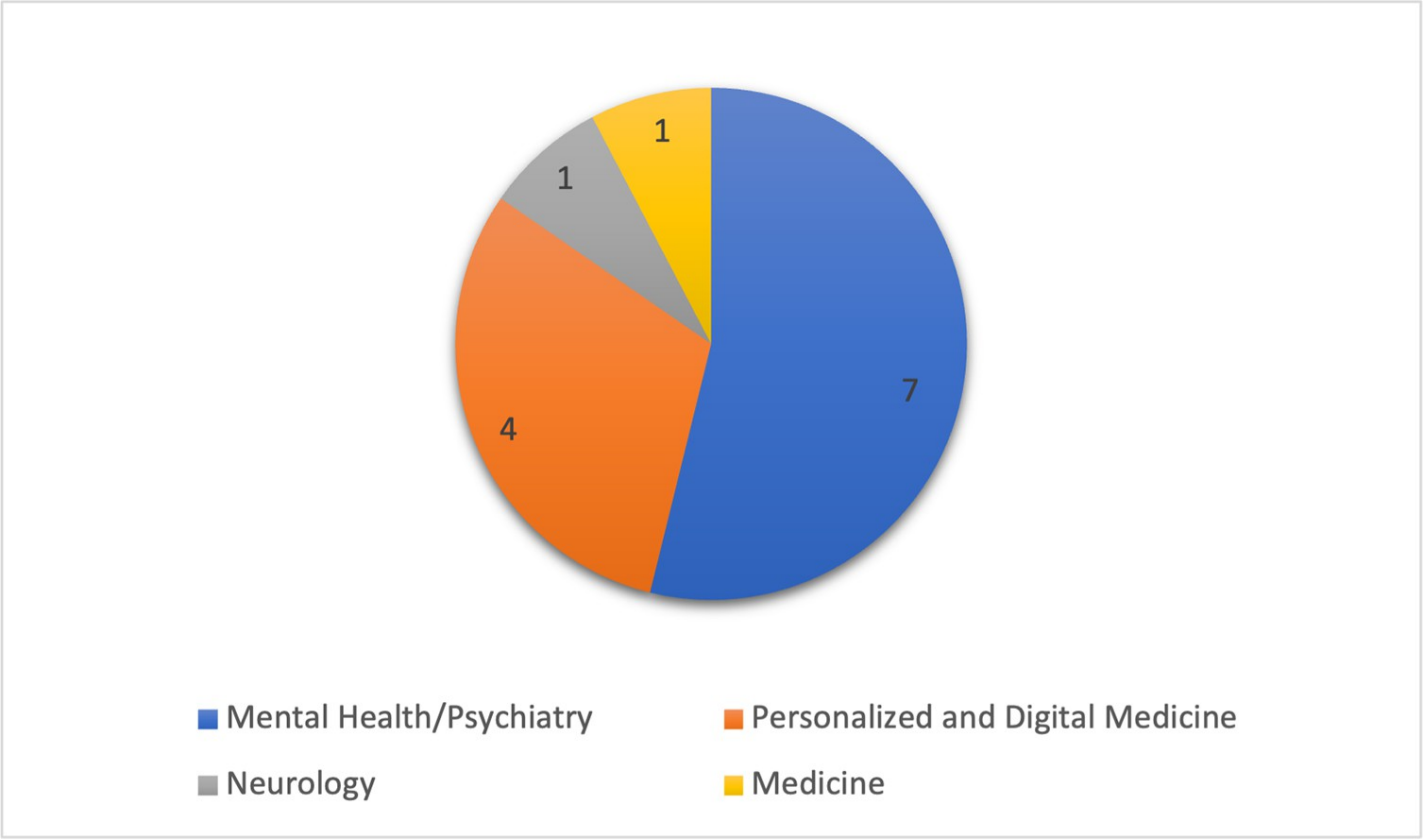
Field area of included articles.

All retrieved papers in our study were published from 2019 onwards, underscoring the novelty of the topic. The publications included in our scoping review encompassed an array of domains, shedding light on the utilization of digital biomarkers in various fields. The largest number of papers concentrated on mental health/psychiatry, investigating potential applications of digital biomarkers for diagnosing and treating mental health conditions. Four articles were published in the personalized and digital medicine realm, while one article fell under each neurology and general medicine (Fig 2.)

It is not surprising that a significant portion of the discussion on digital biomarkers has emerged from the area of psychiatry and mental health, as the field has consistently prioritized the exploration of alternative biomarkers, due to the limitations of traditional biomarkers, which are often invasive or financially burdensome.

**Fig 3:**
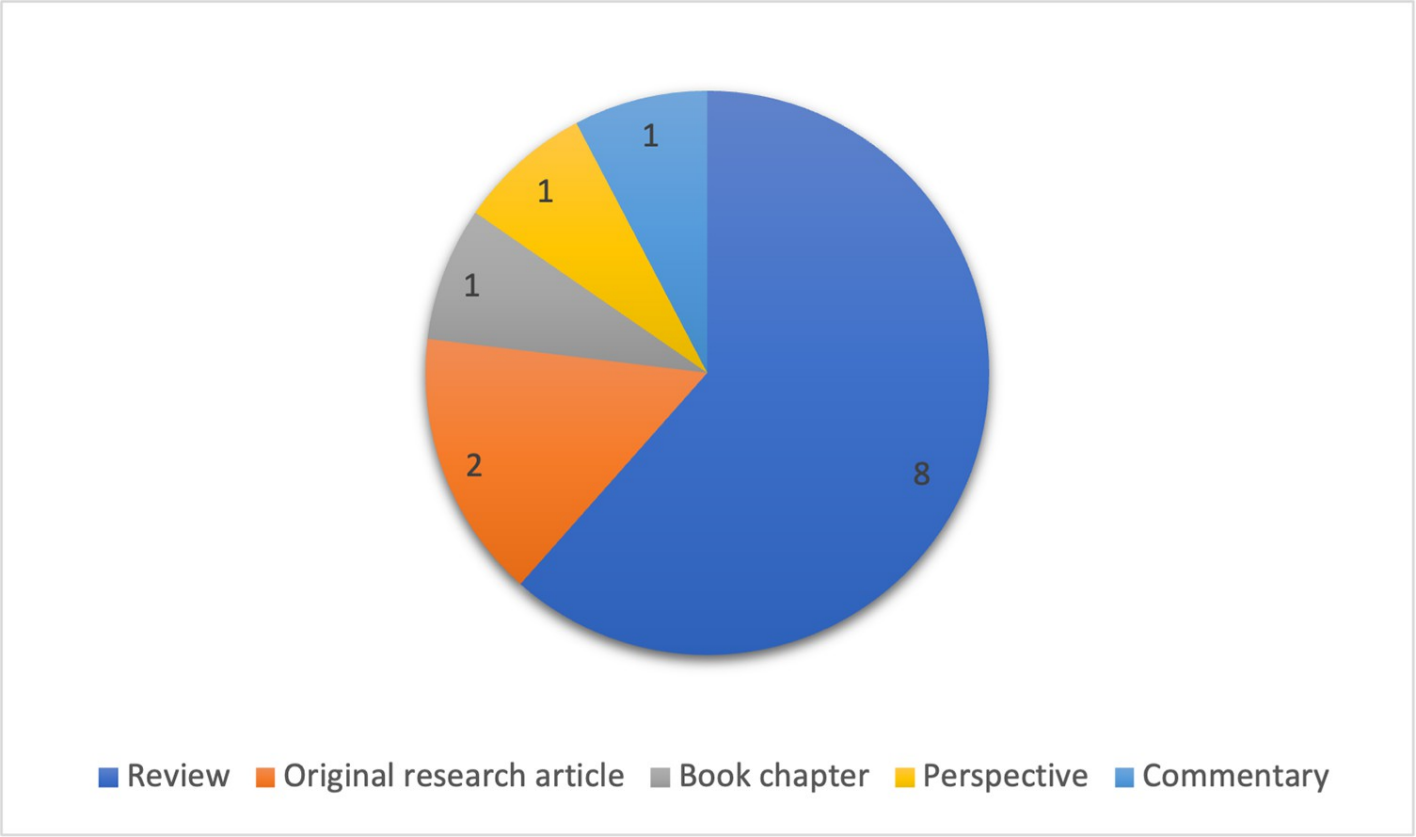
Publication types of included articles.

Most of the articles included in our review are review articles, suggesting that the specific ethical issues related to digital biomarkers so far have remained largely unexplored. Only two papers are labelled as original research, while two are perspective or commentary articles. One record is a book chapter within a specialized publication (Fig 3).

Our review reveals that, within the literature, privacy and data security stand out as central ethical concerns (totaling 13 sources). Closely linked with these concerns is a significant emphasis on the appropriate use of informed consent for acquiring health data through digital technologies (found in six sources). Additionally, the validation of algorithms enabling data analysis, such as risk prediction, is a frequently discussed issue (mentioned in six sources). Furthermore, there are notable considerations regarding equity and sustainability, as reflected in five sources. Transparency, specifically pertaining to data handling and analysis, emerges as a less common yet still significant concern in four articles. Stigmatization and the predictive use of digital biomarkers for incurable diseases also attract ethical attention (discussed in three articles). Ethical worries surrounding data ownership, and regulatory aspects for development and market access, are represented in two sources. Accountability, with regard to the responsible management of data and decision-making processes, is discussed in two sources (Table 2). The following sections further elaborate on our findings, offering concise context and descriptions of the major themes and implications extracted from the retrieved literature.

**Table 2:** Recurrent ethical considerations.

Paper / Topic	Privacy and Security	Informed consent	Equity and Sustainability	Regulation and Guidelines	Transparency	Data Ownership	Validation	Accountability	Impact of stigmatization and Identification of Incurable Diseases
Bourla et al. 2018	x								x
Chen et al. 2022	x	x			x		x		
Coravos et al.2019	x	x	x	x			x		
Dagum & Montag 2022	x	x							
Fröhlich et al. 2022	x			x	x			x	
Gold et al. 2018	x	x		x		x			
Josephy-Hernandez et al. 2021	x	x							
Low 2020	x	x	x						
Parziale & Mascalzoni 2022	x				x	x			x
Sim 2019	x		x		x		x		
Slavich et al. 2019	x						x		
Smith et al. 2021	x						x	x	
Whelan et al. 2022	x		x				x		x

### 4.1 Privacy, data security, and informed consent

Privacy and data security emerge as paramount concerns in the implementation of digital biomarkers, giving rise to ethical dilemmas regarding the protection of sensitive patient data against unauthorized access and misuse [18–30]. The collection and sharing of sensitive personal health information raises questions about who should have access to the data and how it should be used. Patients may hesitate to share data with their healthcare providers, families, and employers due to privacy concerns. They may worry about consequences of disclosing biomarker results, such as stigmatization or potential discrimination by insurance companies or employers, based on health risks revealed by the data.

The use of such data for research purposes and by private companies raises additional ethical concerns. This data is often valuable for commercial purposes, such as targeted advertising, product development, or sale to third parties. Users may be unaware of how their digital biomarker data is being used or may not have sufficient control over its dissemination.

However, in practice, privacy is often difficult to achieve. For instance, Dagum and Montag [21] (drawing on [31]) highlight the challenges of protecting privacy when mobile devices automatically record user whereabouts. Common methods for privacy protection include collecting anonymized data, using third-party storage for anonymized data, and de-identifying identifiable data. However, such strategies might not be effective when dealing with location and accelerometer data obtained through mobile sensors. Many studies have demonstrated that it is in fact possible to identify individuals and their behaviors with a high degree of accuracy based on such anonymized data. Dagum and Montag [21] also emphasize privacy concerns related to data linkage, as combining multiple datasets increases the risk of identification. The simultaneous collection of various types of data from mobile sensors further enhances the probability of linking data to identifying information.

Given the numerous privacy concerns associated with digital biomarkers, ensuring informed consent is essential prior to data collection. The process of informed consent for digital biomarkers requires a setting conducive to good decision-making and should go beyond merely signing a form. Further challenges arise when obtaining consent from individuals with cognitive impairment, such as in cases of neurodegenerative or psychiatric diseases that require special considerations and engagement [23]. This seems to be a particularly urgent issue in digital biomarkers as, so far, they have been employed mostly in such contexts.

### 4.2 Equity and sustainability

Digital biomarkers, if not implemented carefully, can exacerbate existing health disparities, and create further inequities in access and outcomes [20,25,27,30]. One key aspect is the digital divide, with disparities in access to technology and digital literacy preventing marginalized populations from benefiting from digital biomarkers. The economic burden associated with the cost of technologies can further exacerbate the situation. This issue is worsened by the limited availability of mobile health technologies in languages other than English [27], widening the gap between those who have access to the latest healthcare technologies and those who do not, and perpetuating health inequalities. Moreover, acceptance of digital technologies can vary among cultures. As [20] illustrates, in a study promoting physical activity among refugee women, the researcher opted to employ wrist-worn accelerometers to assess daily mobility. These devices were distributed to participants, who were instructed on their usage. Upon returning a week later to collect the data, the researcher discovered that no measurements had been recorded. This outcome was attributed to the cultural and social taboo surrounding the use of wrist-worn mobile technology, as it would have attracted unwanted attention [32].

Additionally, issues arise related to data bias and representation, as development and validation of digital biomarkers often relies on data that does not adequately capture the diverse characteristics of different populations. This can lead to biased algorithms and diagnostic tools that are not equally effective for everyone. Ensuring equity in these tools regarding sex, gender, race, ethnicity, and culture is a significant challenge. As explained in [29], for instance, women are at a higher risk of Alzheimer’s disease (AD), stroke, depression, anxiety disorders, and migraines, factors which increase the risk of dementia. Investigating how gender impacts disease presentation, given the higher prevalence of depression and AD in women, may lead to discovery of distinct digital biomarkers and machine learning techniques for both genders.

Algorithmic fairness in emotional computing should further extend beyond sex and gender disparities. For example, facial analysis algorithms may struggle with faces of diverse racial and cultural backgrounds, due to training on datasets primarily comprising lighter-skinned males [29]. To improve fairness, transparency, and accountability in emotional computing development, both algorithmic and human bias must be addressed.

### 4.3 Validation

The validation of digital biomarkers raises not only epistemological considerations, but also ethical implications that cannot be ignored [19,20,24,27,28,30]. While the primary concern is ensuring the accuracy and reliability of these biomarkers, the moral responsibility to avoid harm is inherently tied to the validation process. Therefore, it is crucial to only make use of digital biomarkers that have undergone thorough validation procedures. Among the different levels of validation of a digital biomarker (verification, validation, analytic validation, clinical validation), the most relevant for end-users is clinical validation [20].

Clinical validation involves assessing whether the measurement of interest accurately reflects the intended concept, such as the patient’s subjective experience, functional ability, or overall well-being. It requires evaluating whether a specific measure, such as gait speed, effectively captures relevant aspects of how a particular patient population feels, functions, or survives, a common task in the validation of biomarkers. A further key aspect of validation is consideration of potential risks and harms associated with false positives, false negatives, or misinterpretation of biomarker results. However, determining how to effectively test and validate digital biomarkers to prevent unnecessary harm resulting from errors is a complex and challenging endeavor.

### 4.4 Regulatory issues

The absence of clear and *ad hoc* regulatory guidelines is widely recognized as a significant ethical concern within the realm of digital biomarkers. This gap in regulation poses a risk of unproven digital biomarkers being brought to market, facilitated by the low barriers to entry [20,22,23]. Currently, the FDA’s oversight of digital biomarkers is embedded within its broader regulation of medical devices and digital health technologies. Typically, digital biomarkers get regulatory approval under the classification of "software as a medical device" (SaMD). SaMDs can fulfill a medical function autonomously, without requiring integration into hardware. However, as digital biomarkers increasingly rely on AI/ML for data interpretation, the regulatory landscape becomes more intricate. There is currently a substantial "regulatory gap" concerning the clinical application of AI/ML, a matter under active discussion with the situation evolving rapidly [16]. This regulatory ambiguity raises ethical questions about patient safety, the reliability of diagnoses, and the potential for exploitation by companies seeking to capitalize on the burgeoning digital biomarker market.

### 4.5 Impact of stigmatization and identification of incurable diseases

Particularly in the field of mental health, concern that the use of digital biomarkers could do more harm than good is a significant ethical dilemma. In practice, these tools can lead to heightened anxiety in patients, or unnecessary treatment due to overdiagnosis. The identification of cases with mild or no symptoms may not only cause distress to individuals, but also burden healthcare systems, including when treatment options are limited or non-existent. Disclosure of "sensitive" data concerning medical conditions or symptoms can adversely affect patients’ lives, giving rise to concerns about social stigma and discrimination.

The acceptability of delegating health monitoring tasks to a machine is also seen as ethically questionable, as patients may associate the use of digital devices (e.g., wristbands) with intrusive forms of monitoring that impact individual autonomy and freedom, similar to electronic ankle tagging of prisoners [18].

### 4.6 Transparency, accountability, and data ownership

Further significant concerns relate to lack of transparency [19,22,27] in the algorithms and methodologies employed to derive digital biomarkers, as well as the allocation of data protection duties and responsibilities among the actors involved in the process [26].

These issues are closely related to the way digital health tools work, and their accountability, including responsibility and liability. While AI and ML are increasingly utilized in tools such as digital biomarkers, there remains a lack of mechanisms for accountability. AI/ML systems are frequently described as operating inside a "black box," meaning that their inner logic and specific decisions are challenging–if not impossible–for humans to elucidate or understand.

Additionally, the introduction of AI into healthcare may have an impact on the liability of doctors. If doctors are directly involved in patient care, but their treatment decisions incorporate recommendations from AI that are not easily explainable, doctors may face challenges concerning their moral and legal responsibilities. This shift in responsibility could eventually impact the trust patients have in healthcare providers and institutions [22].

Lastly, the issue of data ownership and control is ethically sensitive, particularly in research environments [26], where it is often unclear whether data primarily belong to research participants, to researchers, or to study sponsors [23].

## 5. Discussion

### 5.1 Theoretical contributions

Digital biomarkers are expected to transform the way we monitor and manage health, to drive the development of novel paradigms such as precision medicine, and to contribute to preventive medicine through active monitoring and helping people adopt and maintain healthier lifestyles, reducing disease burden at a population level. They also hold great promise to boost clinical research and drug discovery, including the use of digitally measured endpoints in decentralized clinical trials [33].

Considerable attention is being devoted to the ethical and societal aspects of the ongoing digital transformation of medicine. In the space of digital biomarkers, challenges fall into two broad categories: issues related to the digital infrastructure needed to develop, test, and use digital biomarkers; and issues related to specific uses of digital biomarkers. Our findings indicate that infrastructure issues are not fundamentally distinct from the ethical concerns commonly associated with digital health in general [34,35].

In a broad literature review, Tomicic et al. explored the ethical, legal, and social issues (ELSI) associated with monitoring, quantification, and tracking practices mediated by digital devices, including (but not limited to) digital biomarkers [36]. This study identifies several key ethical challenges, including privacy issues, insufficient research, concerns regarding consent, potential impact on human psychology, surveillance, and data security. To a considerable extent, these concerns are related to the level of trust that users place in access and platform providers to ensure secure service environments [37]. Additionally, trust in government bodies and regulatory authorities to safeguard the integrity of the digital environment and mitigate negative impacts for individuals and society has emerged as a crucial factor influencing the adoption and future potential of digital phenotyping in healthcare.

Against this backdrop, our findings illuminate the use-specific and context-dependent nature of the ethical considerations of digital biomarkers. As evidenced by the literature, the applications and uses of digital biomarkers are diverse, varying according to the setting in which they are employed. Consequently, the ethical issues associated with digital biomarkers differ widely depending on the intended use (e.g., early detection of a neurodegenerative disease vs. pneumonia). The deployment of digital biomarkers in clinical practice may present different ethical challenges compared with their utilization in clinical trials to streamline the drug testing process.

### 5.2 Potential solutions

In addition to the ethical challenges we have identified, our sample highlights both infrastructure solutions and other specific solutions, the former resonating with current digital health literature. For example, with regard to privacy and data security, since digital biomarker data contains sensitive and personal information about an individual’s health, behavior, and lifestyle, effective security measures must be implemented to safeguard this data and prevent unauthorized parties from accessing or misusing it. Therefore, the implementation of a robust data governance framework with transparent distribution of data security responsibilities among stakeholders, including financial backers, researchers, digital tool operators, healthcare service providers, and biobanks and databanks, emerges as a fundamental prerequisite to safeguard privacy [26].

With regard to informed consent, technological literacy plays a pivotal role in addressing data collection and privacy concerns in the realm of digital medicine. For instance, as highlighted in [20,23,27] the non-profit organization Sage Bionetworks has pioneered accessible informed consent templates for mobile devices, which can serve as a valuable blueprint.

To promote inclusivity, diversity, and fairness in the development and implementation of digital biomarkers, bias mitigation is crucial, both in training and validating datasets, and in ensuring that digital biomarkers perform evenly across patient groups. This necessitates the involvement of diverse populations in research and development, the mitigation of algorithmic biases, and consideration of the unique needs and contexts of different communities to prevent disparities in healthcare access and outcomes [20,27].

To increase transparency and allow accountability, the establishment of well-defined regulatory guidelines and standards is essential, with regulatory bodies and professional organizations playing a pivotal role. This involves publication of validation studies, disclosure of performance metrics, and sharing of algorithm details, such as the testing properties of digital biomarkers (sensitivity, specificity, and thresholds for action). Increased transparency not only would enhance the scientific credibility and clinical utility of digital biomarkers, but also would foster trust and empowers informed decision making for both healthcare providers and patients.

### 5.3 Practical challenges

Viewing digital biomarkers through the lens of population screening offers a perspective on the interconnected ethical and practical considerations. For example, when considering digital biomarkers as a screening tool for detecting dementia in the general population, specific ethical issues have been noted [38]. Here, the ethical principles of screenings outlined by Wilson and Junger, in their influential 1968 report for the World Health Organization [39], can provide guidance for navigating the complex ethical terrain inherent in the use of these innovative tools. Wilson and Junger’s principles incorporate factors such as the severity of a health issue targeted by screening, accessibility of diagnostic technologies, and availability of accepted treatments [38]. In the context of digital biomarkers, these principles underscore the need to critically assess the potential benefits, risks, and societal implications of digital biomarker-based screening programs to ensure their alignment with the overarching goal of promoting well-being and achieving meaningful healthcare outcomes.

### 5.4 Future research

Considering the early stage of the field of digital biomarkers, it is crucial to conduct further research and engage in ethical discussion to address the issues identified in the literature. Our review serves as a starting point for such discussion and highlights the numerous gaps in existing research. Additionally, it emphasizes the need for a domain- and context-specific ethical analysis of digital biomarkers, taking into consideration factors such as privacy, consent, algorithm bias, equitable access, and validation. Recognition of these issues in the scientific literature underscores the value of ongoing exploration and deliberation, to promote responsible and ethical use of digital biomarkers in healthcare.

It is worth noting that the literature included in our scoping review generally provides limited in-depth discussion on ethical issues related to digital biomarkers. In most of the retrieved papers, ethical concerns were mentioned briefly or listed without further exploration. This observation aligns with the fact that many of these publications primarily focus on scientific aspects rather than ethical considerations. As scientific journals prioritize scientific findings and methodologies, the limited emphasis on ethical discussion is therefore not surprising. However, the recognition of ethical aspects within these papers underscores the need for interdisciplinary collaboration between the fields of bioethics and digital health. By bridging these disciplines, we can foster a more comprehensive understanding of the ethical challenges and promote the integration of ethical frameworks for the development, implementation, and regulation of digital biomarkers.

Further specialized research in the domain of bioethics is arguably needed not only to identify ethical challenges, but to offer guidance linked to specific uses of digital biomarkers. More specifically, guidelines regarding the ethical aspects of digital biomarkers should take into account the specific ethical issues presented by use of digital biomarkers in different medical specialties and clinical settings. Instead of relying solely on overarching principles and generic ethical claims about, for instance, privacy, data security, or informed consent, it may be useful to develop domain- and context-specific guidelines to serve as intermediate-level maxims. Therefore, we argue that to offer support to the clinical development and implementation of digital biomarkers, general frameworks of norms and ethical principles in digital health should be specified into more concrete guidelines for both researchers and healthcare practitioners [40].

### 5.5 Limitations

The main obstacle we encountered in our research was the absence of a widely shared definition of digital biomarkers. Often, the term is used in a broad sense, encompassing the collection of digital data that may not necessarily serve as (bio)markers. While it is true that many ethical issues discussed in relation to digital data collection (e.g., digital phenotyping) may apply to digital biomarkers, it is possible that digital biomarkers have their own specific ethical concerns. However, our findings suggest that these concerns may remain largely concealed, probably in part due to this terminological confusion.

Because of the limited availability of specialized literature on the subject, we were required to adopt a more inclusive approach when selecting papers for our review. This choice allowed us to identify relevant papers across different journals and disciplines, whose quality and scope varies widely. However, it also limited the level of automated screening, and required subjective judgment in determining which papers to include in the final analysis. As a result, the exact replicability of our review may be compromised, as different researchers might make different judgments. Nonetheless, we believe that this issue does not impact on our general findings.

Conversely, we deliberately chose not to include publications on the ethics of digital health or digital phenotyping. Since these were not the primary focus of our review, we made the decision to narrow our scope and concentrate on the specific domain of digital biomarkers, to capture their specific ethical issues.

## 6. Conclusion

So far, there have been few attempts to investigate the ethical issues surrounding digital biomarkers. This is likely because it is an emerging and rapidly evolving field. Additionally, the literature appears to be flawed due to a terminological confusion between digital phenotyping and digital biomarkers. A lack of clear and widely adopted definitions has probably hindered streamlined investigation. Furthermore, the discussion of ethical issues in the broader realm of digital health has likely overshadowed a more detailed analysis of specific technologies such as digital biomarkers. Considering the potential impact of digital biomarkers on healthcare, a more focused and thoughtful ethical reflection is necessary. This scoping review can serve as a starting point for future analyses and investigation.

## Supporting information

S1 Checklist PRISMA-ScR Checklist. (DOCX)
